# The role of the gut microbiome in antibiotic-driven antimicrobial resistance

**DOI:** 10.3389/fmicb.2026.1856738

**Published:** 2026-06-03

**Authors:** Gargi Joshi, Smriti Rani, Dipti Bharti, Namrata Panda, Prerana Chavan, Shalini Mathpal, Sudha Ramaiah, Anand Anbarasu

**Affiliations:** 1 Department of Bio-Medical Sciences, School of Biosciences and Technology, Vellore Institute of Technology (VIT), Vellore, Tamil Nadu, India; 2 Department of Bio-Sciences, School of Biosciences and Technology, Vellore Institute of Technology (VIT), Vellore, Tamil Nadu, India; 3 Medical and Biological Computing Laboratory, School of Biosciences and Technology, Vellore Institute of Technology (VIT), Vellore, Tamil Nadu, India; 4 Department of Biotechnology, School of Biosciences and Technology, Vellore Institute of Technology (VIT), Vellore, Tamil Nadu, India

**Keywords:** antimicrobial resistance, dysbiosis, gut microbiome, gut resistome, horizontal gene transfer

## Abstract

Antimicrobial resistance (AMR) is one of the most pressing threats to global health system. The human gut harbors a complex microbial ecosystem coordinated through mechanisms of metabolic interdependence. The gut microbiota plays a vital role in normal growth and physiological processes of the human body. It serves both as a target of antibiotic-mediated disruption and as a reservoir for the propagation of antimicrobial resistance genes. Although antibiotics remain indispensable for the treatment of bacterial infections, their broad ecological impact on the gut microbiota can undermine the microbial balance that protects the host against pathogen invasion and metabolic dysfunction. The gut microbiome also functions as a reservoir of antimicrobial resistance genes collectively termed the “resistome,” which can be mobilised and transferred between commensal and pathogenic bacteria via horizontal gene transfer mechanisms such as conjugation, transformation, and transduction. This review examines the composition and functions of the human gut microbiota, the mechanism of antibiotic-induced gut dysbiosis, and the role of host factors like age, genetics, diet and immune status, on microbiome dynamics and AMR development. We further evaluate emerging methods for resistome characterisation, which include PCR, next-generation sequencing, functional metagenomics and artificial intelligence-driven tools. Finally, we discuss microbiome-targeted therapeutic strategies such as faecal microbiota transplantation (FMT), phage therapy, CRISPR-based therapies, and antimicrobial peptides for combating AMR and restoring gut microbial homeostasis. Overall, this review highlights that maintaining and re-establishing the integrity of the gut microbiome should be considered a fundamental component of antimicrobial stewardship strategies aimed at controlling AMR worldwide.

## 1 Introduction

Antibiotics have significantly advanced modern medicine by enabling the effective treatment of infectious diseases and supporting advanced medical interventions such as surgery, cancer chemotherapy, and organ transplantation. However, their clinical utility is increasingly being undermined by the rapid emergence and global dissemination of antimicrobial resistance (AMR), which has evolved into a critical threat to global public health. AMR is now recognised as a multifactorial and escalating global crisis caused by the improper use of antimicrobials, inadequate infection control practices, and the limited development of new therapeutics (Holmes et al., 2016; Prestinaci et al., 2015; Tang et al., 2023).

Several studies provide valid evidence of the growing burden of AMR. The worldwide research on antimicrobial resistance (GRAM) project estimated that bacterial AMR directly accounted for nearly1.27 million deaths and was linked with 4.95 million deaths globally in 2019. The latest investigation reveals that, in 2021 alone, around 1.14 million deaths were directly due to AMR. A total of 4.71 million deaths were associated with resistant infections, highlighting the severity and widespread consequences of AMR (GBD 2021 Antimicrobial Resistance Collaborators, 2024).

Moreover, current findings indicate a significant increase in multidrug resistance (MDR) infections, with a universal growing surge of over 40% in the medical establishments (Marino et al., 2025). The widespread and repeated use of antibiotics use can reduce microbial diversity. This can eliminate basic commensal taxa, and produce ecological niches that dilate the expansion of resistant organisms and opportunistic pathogens (Zhang and Hochwald, 2016). Importantly, the gut microbiome serves as a reservoir of AMR genes, collectively known as the ‘resistome’. These resistance genes can spread between commensal and pathogenic bacteria through horizontal gene transfer (HGT) mechanisms, including conjugation, transformation, and transduction (von Wintersdorff et al., 2016; McInnes et al., 2020).

The latest metagenomic research has indicated that even short-term antibiotic use may lead to modifications in the resistome and microbial community structure, with recovery often being partial or delayed (Dethlefsen and Relman, 2011). Crucially, disorganisation of the gut microbiota has been associated with increased susceptibility to infections such as *Clostridioides difficile,* as well as increased colonisation by resistant pathogens, including *Enterococcus* and *Enterobacteriaceae* species (Taur et al., 2012; Buffie and Pamer, 2013). Thoroughly, this strengthens the need for microbiome-conscious therapeutic strategies, including targeted antimicrobial use, microbiota re-establishment approaches, and the development of narrow-spectrum or non-disruptive antimicrobials to lessen AMR progression. AMR arises when bacteria are exposed to antibiotics. Such resistance bacteria can produce enzymes that inactivate antibiotics, alter drug targets, reduce membrane permeability, or actively pump antibiotics out of the cell (Blair et al., 2015). In clinical settings, this results in infections that are more difficult to treat, require prolonged hospitalisation, and often necessitate the use of more toxic or expensive last-line therapies (Laxminarayan et al., 2013).

The evolution and expansion of AMR are primarily operated by the misuse and overuse of antibiotics in humans. This includes improper prescription of antibiotics for viral infections, inappropriate broad-spectrum therapy, and incomplete medications courses (Ventola, 2015). Simultaneously, the extensive use of antibiotics in livestock for growth promotion and disease prevention contributes to the emergence of resistant bacteria. These bacteria can spread to humans through the food chain, direct contact, and environmental exposure (Marshall and Levy, 2011).

Inside the human body, the gastrointestinal tract harbors the largest and most complicated microbial ecosystem, known as the gut microbiome. This diverse community of microorganisms plays an essential role in the host health by promoting digestion and metabolism, improving and regulating the immune system, and protecting against invading pathogens (Sommer and Bäckhed, 2013). By competing with harmful microorganisms for nutrients and ecological niches, the gut microbiome provides colonisation resistance and contributes to intestinal homeostasis (Buffie and Pamer, 2013). Through these mechanisms, a healthy gut microbiome works as a vital line of defence against infections and diseases.

Although antibiotics are essential for treating bacterial infections, they are also among the most potent disruptors of the gut microbiome. Antibiotic administration gives rise to dysbiosis, loss of resident microbiota, and the spread of opportunistic or pathogenic organisms. These disruptions may persist long after treatment, especially after continuous antibiotic courses (Dethlefsen et al., 2008). Antibiotic associated dysbiosis has been linked to diarrhea, increased vulnerability to opportunistic infections such as *Clostridioides difficile,* immune dysregulation, and metabolic disruption (Jernberg et al., 2010; Becattini et al., 2016).

Such ecological disruptions deplete colonisation resistance (CR) create appropriate conditions for resistant organisms to expand (Buffie and Pamer, 2013).

Above all, the gut microbiome also acts as a source of AMR genes. Exposure to Antibiotics lead to the increase in the abundance of resistant strains and facilitates HGT between commensal and pathogenic bacteria. This allows rapid dispersion of resistance linked features both within the gut ecosystem and beyond (Smillie et al., 2011). Consequently, the gut functions not only as a target of antibiotic-mediated damage but also as a core site for the amplification and dissemination of AMR (Sommer et al., 2009; van Schaik, 2015).

Despite increasing recognition of the gut microbiome’s role in AMR, the long-term consequences of antibiotic-induced dysbiosis and the influence of host-related factors such as diet, age, immune status, hospitalisation, and geographic context remain incompletely understood (Yatsunenko et al., 2012; David et al., 2014). Moreover, current clinical approaches often overlook microbiome preservation and restoration as essential component of antimicrobial stewardship (Langdon et al., 2016). This review addresses these gaps by integrating current evidence on gut microbiome composition and function, the mechanisms by which antibiotics promote AMR in the gut, and emerging strategies to mitigate resistance while preserving microbial health.

## 2 Gut microbiome: composition and protective role

The gut microbiome of human represents a complicated and diverse ecosystem composed of viruses, bacteria, archaea, and fungi, whose taxonomic composition differs markedly among individuals and is influenced by several determinants such as age, environmental factors and host genetics. A large-scale analysis involving 18,340 subjects showed that only 9 out of 410 identified bacterial genera were present in more than 95% of samples, highlighting substantial variability between individuals and host genetic factors, such as the lactase (LCT) locus, have been associated with variations in *Bifidobacterium* abundance (Kurilshikov et al., 2021).

The major bacterial phyla found in the healthy human gut are *Firmicutes* and *Bacteroidetes*, followed by *Actinobacteria, Proteobacteria, Verrucomicrobia*, and *Fusobacteria*. Important commensal genera, including *Bacteroides, Faecalibacterium, Bifidobacterium, Roseburia, Lactobacillus*, and *Akkermansia*, contribute significantly to intestinal homeostasis through nutrient metabolism, immune modulation, and maintenance of epithelial barrier integrity (Sommer and Bäckhed, 2013; Kho and Lal, 2018). Among these, *Faecalibacterium prausnitzii* and *Roseburia* species are important producers of short-chain fatty acids (SCFAs), particularly butyrate, which serves as a primary energy source for colonocytes and exerts anti-inflammatory effects within the intestinal mucosa (Parada Venegas et al., 2019).

Age acts as a major factor shaping gut microbiota composition. Studies in healthy Vietnamese individuals found that children under 2 years of age possessed a unique gut microbiota and carried a higher abundance of AMR genes compared with older children and adults, which suggests that early childhood may be a critical period for the development of gut microbiota and its associated health implications (Pereira-Dias et al., 2021). Comparable age-associated changes in microbial taxa, such as *Bacteroides, Firmicutes* and *Proteobacteria,* as well as changes in functional gene content, have been observed among Chinese individuals at different life stages (Wu et al., 2021).

Beyond its compositional diversity, the gut microbiome contributes to host protection not only through its composition but also through immune modulation, barrier maintenance and metabolic regulation. Experimental studies in mice showed that depletion of gut microbiota worsened *Streptococcus pneumoniae* infection by promoting bacterial dissemination, inflammation, tissue damage and mortality. Restoration of the microbiota through faecal microbiota transplantation normalised lung bacterial levels, balanced cytokine responses and improving macrophage phagocytic capacity, thereby highlighting a protective role of gut-lung axis (Knapp, 2016). Studies using a dextran sulfate sodium colitis model demonstrated that extracellular vesicles from *Akkermanisa muciniphila* were shown to reduce epithelial IL-6 production, improve clinical symptoms, including weight loss, colon shortening and inflammatory cell infiltration, highlighting their role in protecting the intestinal mucosa (Kang et al., 2013).

The gut microbiota also plays an important role in protecting the host against non-infectious stressors. In a *Caenorhabditis elegans* model, microbiome restructuring enriched neomycin-resistant bacteria carrying detoxifying enzymes, which protected the worms from antibiotic toxicity. However, this protective shift also negatively affected metabolic functions and resistance to infection (Kim et al., 2025). Overall, evidence from experimental and human studies demonstrates that the gut microbiome is highly dynamic and that commensal microorganisms, together with their metabolites, provide essential protection against pathogens, toxic compounds, and inflammatory damage (Kurilshikov et al., 2021).

## 3 The gut as a major reservoir of antimicrobial resistance genes

Metagenomic studies consistently demonstrate that the human gut serves as a significant reservoir of antimicrobial resistance genes (ARGs), collectively referred to as the gut resistome (Kent et al., 2020). A study of 246 healthy individuals from a Chinese longevity region revealed 606 antimicrobial resistance genes, with older participants exhibiting the highest abundance and diversity. The tetracycline resistance gene *tetQ* was the most prevalent, and Bacteroides was the main carrier of ARGs (Wu et al., 2021).

High-resolution analyses of infant microbiomes show that the gut resistome is already well established early in life. Monthly sequencing of 12 healthy infants revealed a core group of ARGs primarily consisting of efflux-related genes commonly connected with *Enterobacteriaceae*, while the overall abundance of ARGs decreased throughout the first year as Proteobacteria levels declined (Kim et al., 2025). A large-scale study analysing 4,132 metagenomes from 963 infants across six countries identified 4,285 ARGs. It showed that ARG abundance decreased as infants aged during the first 3 years, with *Escherichia coli* (*E. coli*) carrying a disproportionate share of these genes, while the gradual accumulation of ARGs indicated increased horizontal gene transfer (HGT) within the developing gut microbiota (Xu et al., 2024).

Mechanistic studies using *in vivo* models have demonstrated efficient transfer among gut microbes through plasmid-mediated transfer. In a mouse model with transplanted human microbiota, an RP4 conjugative plasmid carrying multiple ARGs was shifted from engineered *E. coli* donors to recipient families such as *Lachnospiraceae*, *Clostridiaceae* and *Pseudomonadaceae*, where transconjugant strains persisted within the community (Sher et al., 2025). Hi-C-based mapping in neutropenic transplant patients identified extensive HGT networks in the gut microbiome, where mobile ARGs were associated with a wider variety of microbial taxa than in healthy controls, implying that antibiotic-exposed gut environments favour increased gene transfer among resident microbes (Kent et al., 2020). Clinical and environmental evidence highlights that gut commensals act as a significant reservoir of ARGs. In healthy Egyptian individuals, 64.3% of commensal *E. coli* isolates exhibited multidrug resistance and carried various plasmid-mediated ARGs associated with resistance to beta-lactam, sulfonamide, tetracycline, quinolone and aminoglycoside antibiotics (Tawfick et al., 2022). Collectively, the available evidence indicates that the gut microbiota contains abundant mobile resistance genes that can move among commensal microbes and potentially be acquired by pathogens, emphasising its significance as an AMR reservoir (Sher et al., 2025).

## 4 Antibiotic exposure and gut dysbiosis

Antibiotic use is a primary cause of gastrointestinal disturbances, that lead to a condition known as gut dysbiosis. This condition is characterised by reduced microbial diversity, structural change, and loss of microbial function (Ramirez et al., 2020). Even though antibiotics are important for controlling pathogenic infections, their non-selective activity can disrupt commensal microbial communities, thereby compromising intestinal homeostasis (Cusumano et al., 2025). Recent evidence suggests that antibiotic-induced changes in the gut microbiota can persist for extended periods, potentially contributing to immune dysregulation, metabolic imbalance, and increased susceptibility to opportunistic infections (Bernabè et al., 2023; Lathakumari et al., 2024; Carías Domínguez et al., 2025). Importantly, the extent and duration of antibiotic-induced dysbiosis are affected by the type of antibiotic, duration of treatment, and host-specific factors such as age, diet, and composition of microbiota. All together, these findings highlight antibiotic exposure as a critical modulator of host-microbiome interactions with both short and long-term physiological consequences (Donald and Finlay, 2023).

### 4.1 Definition and overview of gut dysbiosis

**Figure 1:**
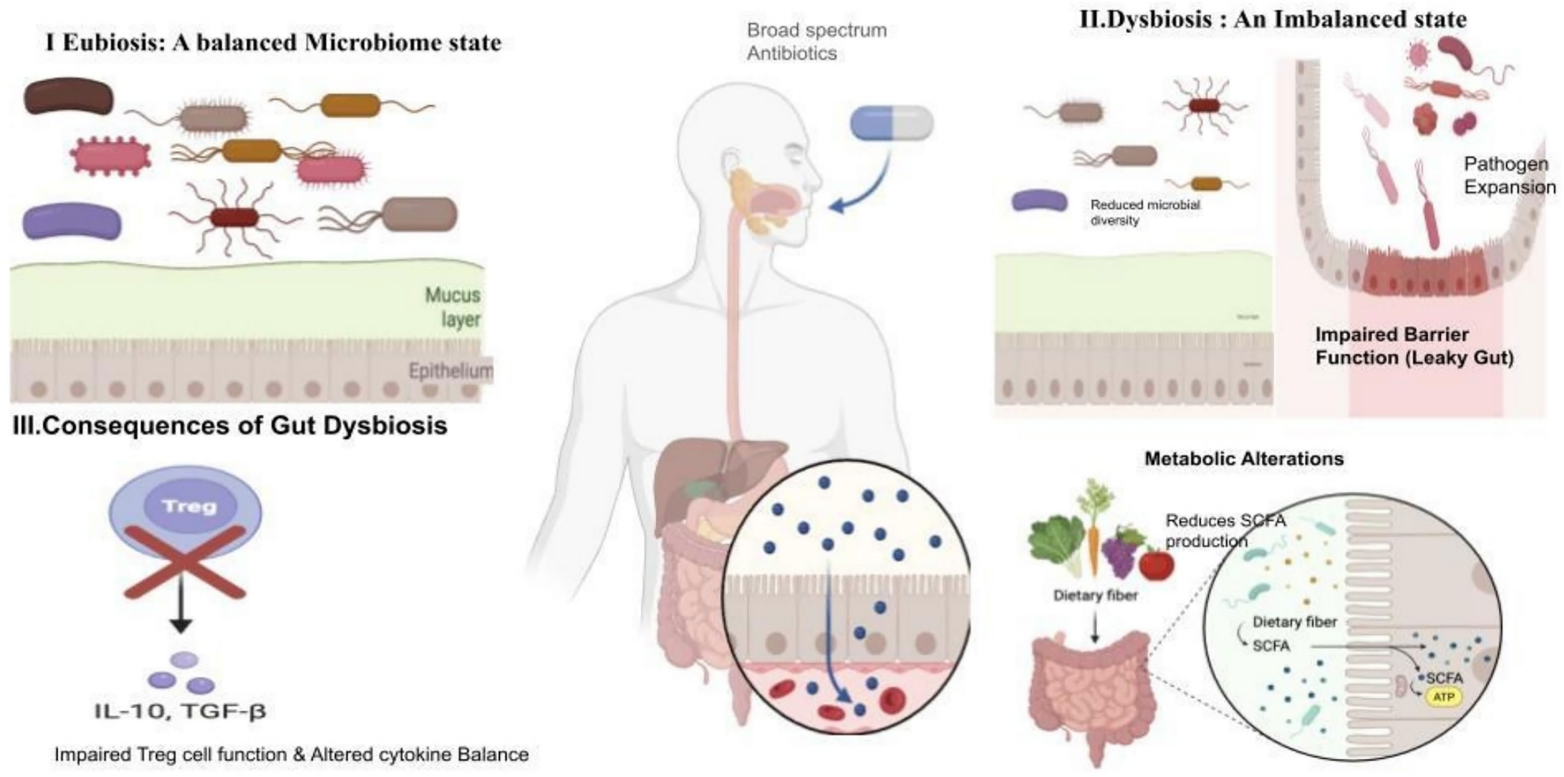
Antibiotic-induced gut dysbiosis and its multidirectional consequences. Broad-spectrum antibiotic exposure disrupts the healthy gut microbiome, reducing microbial diversity and promoting pathogen expansion. This dysbiotic state impairs intestinal barrier function, alters immune responses, and decreases short-chain fatty acid (SCFA) production, ultimately contributing to inflammation and metabolic dysfunction. Created with BioRender.com.

Gut dysbiosis is characterised by an imbalance in the composition, diversity, and functional capacity of the intestinal microbiota relative to a host-adapted homeostatic baseline (Lin and Medeiros, 2023). Figure 1 shows the antibiotic-induced gut dysbiosis and its associated consequences. Due to substantial inter-individual variability, there is no universal definition of a healthy microbiome. However, a balanced gut ecosystem is typically dominated by the phyla *Firmicutes* and *Bacteroidetes*, which play essential roles in maintaining metabolic and immunological homeostasis (Ianiro et al., 2022; Meng et al., 2020). Under physiological conditions, these microbial communities contribute to the metabolism of complex carbohydrates, proteins, and lipids (Fusco et al., 2023) and facilitate the production of SCFAs, including acetate, propionate, and butyrate (Kimura et al., 2020). SCFAs are particularly important for gut health, with butyrate serving as the primary energy source for colonocytes and supporting epithelial barrier integrity through tight junction regulation (Liang et al., 2022). In addition, the gut microbiota modulates host immune responses through AMP production and cytokine signalling pathways (Meng et al., 2020). Dysbiosis is typically characterised by reduced alpha diversity (e.g., Shannon and Chao1 indices), depletion of beneficial commensals, and expansion of opportunistic taxa such as *Proteobacteria* (Li et al., 2025). Among the various external factors influencing gut microbial composition, antibiotics are considered one of the major drivers of dysbiosis, often inducing rapid and profound ecological shifts within the gut microbiome (Laubitz et al., 2021).

### 4.2 Impact of antibiotics on gut microbiota

The magnitude and duration of antibiotic-induced microbiota disruption largely depend on the spectrum of activity, duration of exposure, and pharmacokinetic properties of the antibiotic. Broad-spectrum antibiotics, including carbapenems and piperacillin-tazobactam, exert more pronounced effects than narrow-spectrum agents due to their comprehensive depletion of obligate anaerobic commensals, especially SCFA-producing bacteria. The impact of antibiotics on gut microbiota is summarised in Table 1 (Ng et al., 2019; Peto et al., 2025). Antibiotic exposure typically results in a rapid decline in microbial diversity, loss of functional redundancy, and expansion of opportunistic taxa, especially *Proteobacteria* (Bidell et al., 2022). Experimental and clinical studies have demonstrated that even short-term antibiotic use can significantly reduce microbial diversity within days, whereas prolonged or repeated exposure may result in long-lasting or incomplete recovery. For example, ceftriaxone treatment in murine models has been shown to suppress diversity indices including Chao1 and Shannon indices, for up to 14 months post-treatment, indicating a sustained loss of microbial resilience (Laubitz et al., 2021; Winters et al., 2024). At the compositional level, antibiotics induce selective shifts in microbial populations. Broad-spectrum agents reduce dominant phyla such as *Firmicutes* and *Bacteroidetes*, while facilitating the expansion of facultative anaerobes (Magne et al., 2020). Specific antibiotics also exert differential effects; for instance, amoxicillin has been associated with an increase in *Prevotellaceae*, whereas tetracycline exposure may favour *Streptococcaceae* (Dhariwal et al., 2023). Systematic recovery of microbiota is highly variable. While some microbial taxa may re-establish after treatment cessation, others may remain permanently depleted, leading to an alternative dysbiotic state. This variability highlights a major gap in current research regarding the long-term resilience and restoration of the gut microbiome following antibiotic exposure (Bajinka et al., 2020).

**Table 1:** Impact of different antibiotic exposures on gut microbiota composition and recovery dynamics.

Antibiotic type	Microbial impact	Recovery pattern
Broad-spectrum	Major depletion of commensals, diversity	Slow, often incomplete
Narrow-spectrum	Targeted disruption	Faster recovery
Short-term exposure	Transient diversity loss	Rapid recovery
Long-term exposure	Persistent dysbiosis	Delayed/incomplete

### 4.3 Consequences of gut dysbiosis following antibiotic exposure

The effects of antibiotic-induced dysbiosis extend beyond the gut lumen, affecting systemic metabolic and immune hemostasis. One of the primary mechanistic pathways involves the disruption of SCFA production. Decreased levels of acetate, propionate and butyrate weaken the epithelial barrier integrity by attenuating tight junction proteins, thereby increasing intestinal permeability (Nireeksha et al., 2025). This comprised barrier accelerates the translocation of microbial components such as lipopolysaccharides (LPS), which can trigger systemic inflammation and contribute to the pathogenesis of inflammatory bowel disease (IBD) and metabolic disorders (Caparrós et al., 2021).

Additionally, dysbiosis decreases CR, creating a favourable environment for opportunistic pathogens. A well-established example is *Clostridiodes difficle,* whose colonisation is promoted by antibiotic-induced alterations in bile acid metabolism and microbial competition (Akuzum and Lee, 2022). From an immunological view, dysbiosis disrupts several key regulatory pathways, such as reduced generation of T helper 17 (Th17) cells, an imbalance in regulatory T cells (Treg), diminished production of interleukin-22 (IL22), and a reduction in the anti-inflammatory effects of SCFAs. Collectively, these alterations weaken mucosal immunity and increase susceptibility to infections and chronic inflammatory conditions (Zheng et al., 2020). In addition, antibiotic exposure contributes to the expansion of gut resistome, thereby promoting HGT and the persistence of AMR genes within microbial populations. However, inconsistencies remain regarding the long-term consequences of antibiotic-induced dysbiosis. While some studies report partial microbiota recovery, others suggest persistent functional losses, indicating that host genetics, diet, and environmental exposures may act as key modulators of microbiota recovery and resilience (Shayista et al., 2025).

## 5 Mechanism by which antibiotics promote antimicrobial resistance in the gut

**Figure 2:**
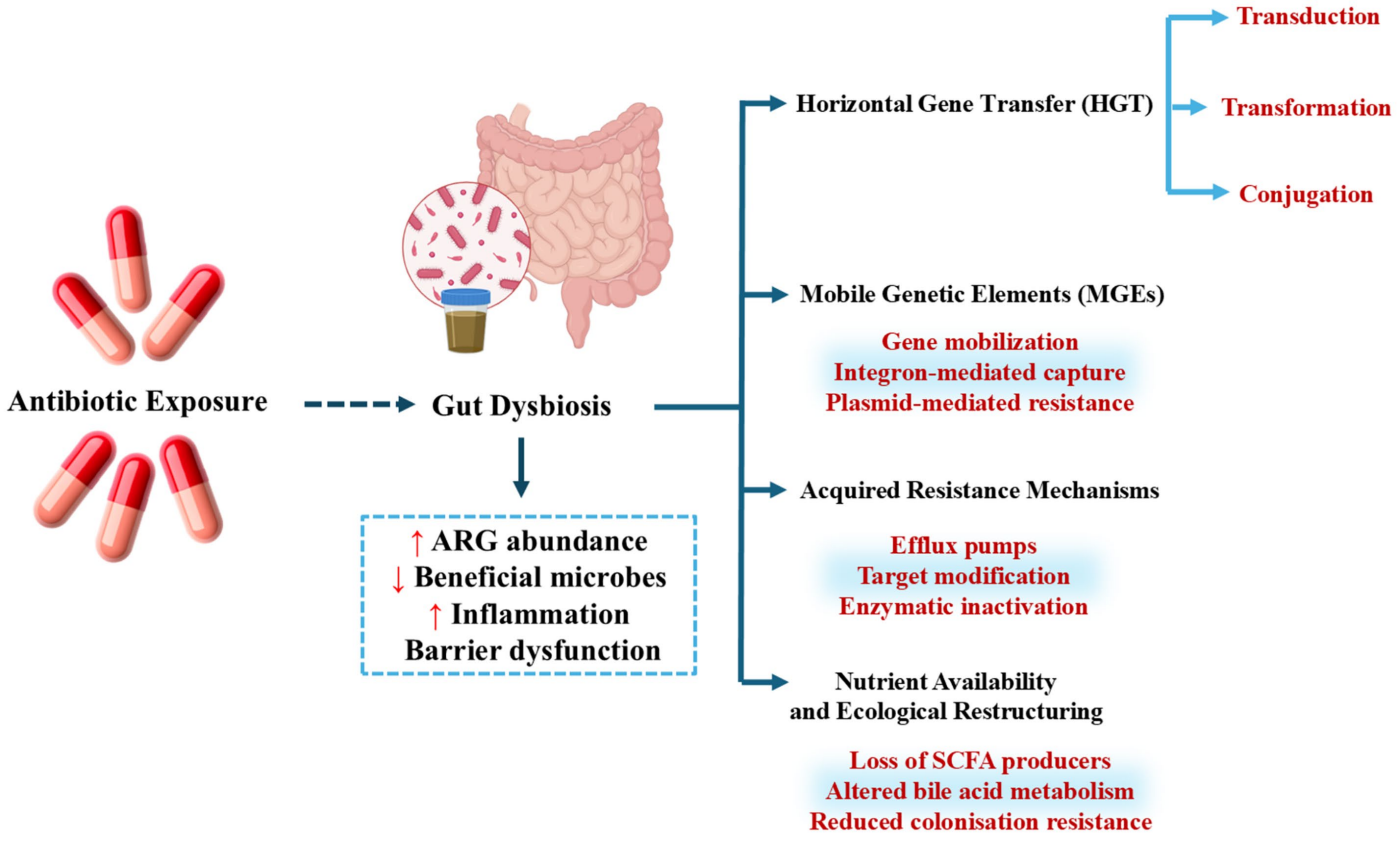
Mechanisms of antibiotic-promoted antimicrobial resistance in the gut.

In humans, the gastrointestinal tract contains one of the most densely populated microbial ecosystems and serves as a major reservoir of ARGs, collectively called the gut resistome. Antibiotic exposure imposes strong selective pressure on the microbiome, causing ecological disruption and evolutionary changes that promote resistant taxa. Even short-term antibiotic use can cause lasting changes in the resistome (MacLean and San Millan, 2019; Fredriksen et al., 2023). Antibiotics cause dysbiosis that reduces colonisation and favours resistant organisms and ARG spreading. Gut AMR emerges from interconnected processes, not a single pathway. These processes simultaneously drive resistome expansion in the gut (Dethlefsen and Relman, 2011; Li et al., 2019). Figure 2 and Table 2 explain the mechanism and its key processes of antimicrobial resistance in the gut.

**Table 2:** Illustrating the interconnected pathways by which antibiotic exposure drives antimicrobial resistance in the gut microbiome, including dysbiosis, enhanced horizontal gene transfer, mobilisation of genetic elements, and ecological niche restructuring.

Mechanism	Key process	Outcome
Dysbiosis	Loss of commensals and reduced diversity	Expansion of resistant taxa
HGT	Transfer of ARGs via conjugation, transformation, transduction	Rapid spread of resistance
MGEs	Mobilisation of plasmids, transposons, integrins	Co-selection and integration of ARGs
Nutrient shifts	Altered metabolic landscape, reduced competition	Selective advantage for resistant bacteria
Acquired mechanisms	Efflux pumps, target modification, enzymatic degradation	Enhanced survival under antibiotic pressure

### 5.1 Horizontal gene transfer (HGT)

HGT is a primary driver of the dissemination of ARGs within the gut microbiome. The major mechanisms include conjugation, transformation, and transduction; among these, conjugation is considered the most efficient and clinically relevant mechanism particularly within the densely populated environment of the gut, where direct cell-to-cell contact facilitates gene exchange (McInnes et al., 2020). Exposure to subinhibitory concentrations of antibiotics triggers stress responses in bacteria such as the activation of the SOS pathway, which can lead to increased DNA uptake, bacteriophage induction, and plasmid transfer (Breidenstein et al., 2012). Collectively, these processes create conditions conducive to the spread of ARGs. However, the relative importance of HGT compared to clonal expansion *in vivo* remains unclear (Baharoglu et al., 2010; Breidenstein et al., 2012).

Transformation involves the uptake of exogenous DNA by bacteria and its incorporation into the bacterial genome. More than 80 bacterial species have been identified as naturally competent, including clinically significant pathogens such as *Neisseria gonorrhoeae*, *Vibrio cholerae,* and *Streptococcus pneumoniae* (Johnston et al., 2014; Blokesch, 2016). All of these are capable of acquiring resistance genes through this process. Although the majority of exogenous DNA is degraded due to DNase activity within the gut, intact plasmid DNA has nevertheless been detected in the intestinal contents of animals, suggesting that transformation may contribute to the dissemination of ARGs within the gut environment (Jonas et al., 2001).

Another potential mechanism of gene transfer involves membrane vesicles (MVs), which are nanoscale structures primarily secreted by Gram-negative bacteria (Gill et al., 2019). These vesicles can transfer proteins, enzymes, and genetic material, including plasmids containing ARGs into recipient cells through membrane fusion. *In vitro* studies conducted on *Acinetobacter* and *Escherichia coli* have demonstrated the potential of MVs in plasmid transfer (Yaron et al., 2000; Rumbo et al., 2011).

Transduction refers to the transfer of bacterial DNA mediated by bacteriophages through generalised, specialised, or lateral transduction mechanisms. A diverse array of phages exists within the human gut including those carrying ARGs and their numbers can increase upon exposure to antibiotics. Experimental evidence suggests that phage-mediated transduction contributes to genetic diversity and the evolution of antibiotic resistance among gut-associated bacteria. However, the overall significance of phage-mediated ARG transfer within the gut microbiome remains to be fully elucidated (Chiang et al., 2019).

Among all HGT mechanisms, conjugation is considered the primary pathway for ARG dissemination within the gut ecosystem. During this process, mobile genetic elements such as plasmids and integrative conjugative elements (ICEs) are directly transferred between bacterial cells (Ilangovan et al., 2015). These mobile elements often harbor not only ARGs but also genes associated with heavy metal tolerance, disinfectant resistance, and other adaptive traits, thereby facilitating co-selection under various environmental pressures (McInnes et al., 2020). The dense microbial community and mucus-rich environment of the intestinal tract provide an ideal setting for conjugative transfer between commensal and pathogenic bacteria. Evidence supporting this includes the detection of vancomycin resistance (*vanB*) transposons in gut commensals such as *Eggerthella lenta* and *Clostridium innocuum*, indicating that these organisms may serve as previously unrecognised reservoirs of ARGs (Stinear et al., 2001). In another clinical study, IncL/M plasmids carrying bla OXA-48 were identified in *Enterobacter cloacae*, *Escherichia coli*, and *Klebsiella pneumoniae* isolates obtained from the same patient, signaling plasmid transfer within the patient’s gut microbiome (Cremet et al., 2012). Furthermore, conjugative plasmids are capable of mobilising otherwise non-self-transmissible DNA, thereby significantly accelerating the dissemination of antimicrobial resistance within the gut microbial community.

### 5.2 Mobile genetic elements (MGEs)

The HGT potential is relies on the mobile genetic elements (MGEs), which acts as the vectors for mobilisation and integration of ARGs. MGEs, which include plasmids, transposons, and integrons, promote both intra-and inter-genomic transfer of resistance determinants, thereby forming the foundation of the structural evolution of the gut resistome (Bargheet et al., 2025).

Plasmids are self-replicating extrachromosomal DNA elements that often carry clusters of MDR gene and are spread via conjugation. Transposons enable the movement of ARGs between plasmids and chromosomal DNA, whereas integrons capture and express gene cassettes through site-specific recombination. These elements often encode clinically significant resistant determinants, such as *β*-lactamases and carbapenemases (Botelho and Schulenburg, 2021).

Additionally, MGEs enable co-selection, a process in which multiple resistance genes linked on a single genetic element are maintained together under selective pressure. Exposure to antibiotic has been linked to the increased abundance and activity of MGEs, thereby increasing the dissemination potential of ARGs within dysbiotic microbial communities (Jiang et al., 2019). However, distinguishing whether MGE enrichment is a direct consequence of antibiotic pressure or a secondary effect of ecological disruption remains an area of ongoing research (Radovanovic et al., 2023).

### 5.3 Nutrient availability and ecological restructuring

Antibiotic-induced changes in the microbial composition also led to significant changes in the metabolic landscape of the gut. The reduction of dominant commensal populations disturbs established metabolic networks and cross-feeding interactions, resulting in the accumulation of previously utilised nutrients (Wollmuth and Angert, 2023). This ecological shuffling creates a favourable habitat for resistant and opportunistic organisms. Increased availability of the host-derived substrates, like mucosal glycans and sialic acid, along with altered bile acid profiles, provides a metabolic advantage to resistant taxa. In addition, the decreased microbial competition following antibiotic exposure allows these organisms to exploit newly available resources and expand in the gut ecosystem (Baquero et al., 2021).

Furthermore, many resistance mechanisms impose a metabolic fitness cost under normal conditions, however, the nutrient-rich environment associated with dysbiosis can offset these costs, enabling resistant bacteria to persist and outcompete recovering commensal populations (Kocsis et al., 2025). Collectively, these findings highlight the role of ecological niche restructuring as a critical, yet often underappreciated driver of AMR persistence (Yang et al., 2025).

### 5.4 Acquired resistance mechanisms

In addition to ecological and horizontal genetic processes, bacteria employ intrinsic molecular mechanisms to survive antibiotic exposure. One of the most prominent mechanisms involves the overexpression or modification of multidrug efflux pumps, which actively expel antimicrobial agents from the cell, thereby reducing intracellular drug concentrations to sub-lethal levels (Li et al., 2023a; Elbaiomy et al., 2025b).

Other key resistance mechanisms include, target site modification, which prevents antibiotic binding (e.g., mutations in gyrase or ribosomal proteins), enzymatic degradation, such as β-lactamase-mediated hydrolysis of antibiotics, and reduced membrane permeability, which limits antibiotic uptake (Elbaiomy et al., 2025a). Sub-inhibitory antibiotic concentrations within the gut can act as both selective and mutagenic pressures, accelerating the emergence of these adaptive traits. Over time, these resistance mechanisms may become established within bacterial populations, particularly when combined with dissemination through mobile genetic elements (MGEs) (Gutiérrez et al., 2021).

## 6 Antibiotic-induced loss of colonisation resistance

### 6.1 Definition of colonisation resistance

Colonisation resistance (CR) refers to the capacity of the indigenous gut microbiota to prevent exogenous pathogens and opportunistic endogenous organisms from establishing themselves, expanding, and causing infection within the gastrointestinal ecosystem (Ducarmon et al., 2019). This multilayered defence results from a complex web of competitive microbial interactions that work in coordination with mucosal immune effectors to maintain a hostile environment for invading pathogens. These interactions include nutrition scavenging, colonisation site occupation, bacteriocin synthesis, and luminal pH control. Commensal obligate anaerobes, particularly those from the families *Lachnospiraceae, Ruminococcaceae*, and *Bacteroidales*, inhibit potential pathogens by utilising essential substrates such as amino acids, simple sugars, and host-derived mucins, thereby establishing nutritional exclusion as a fundamental defence mechanism (Buffie and Pamer, 2013). Secondary bile acids produced by *Clostridiales* provide an additional chemical barrier by inhibiting *Clostridium difficile* spore germination and vegetative growth, although this protective mechanism is highly sensitive to microbiome disruption (Łukawska and Mulak, 2022). Through defined-community transplantation experiments and germ-free animal models, the mechanistic understanding of CR has significantly improved, revealing that specific microbial taxa, rather than overall microbial diversity alone, represent the functionally critical components of CR (Aghighi and Salami, 2024).

### 6.2 Mechanisms of antibiotic-mediated colonisation resistance disruption

Antibiotics disrupt CR through the simultaneous alteration of multiple interconnected pathways, with effects extending far beyond their primary bacteriostatic or bactericidal activity (Fishbein et al., 2023). The most critical anaerobic microbial groups responsible for secondary bile acid production and competitive exclusion for nutrients are eradicated by broad-spectrum beta-lactams, fluoroquinolones, and lincosamides, which collectively strip away several overlapping layers of the CR (Yoon and Yoon, 2018). At the metabolic level, the consequent reduction of butyrate-producing *Firmicutes* jeopardises colonocyte energy supply and mucosal barrier integrity, while the subsequent depletion of secondary bile acids deoxycholic acid and lithocholic acid removes a potent inhibitor of *C. difficile* spore germination (Aghighi and Salami, 2024). The breakdown of fermentative metabolism also releases fucose and host-derived sialic acid from colonocyte-associated glycans, offering alternative carbon sources that give *Salmonella* and *C. difficile* a selective growth advantage in the post-antibiotic gut environment (Buffie and Pamer, 2013). Immunological components of CR are also impaired by antibiotic-induced reductions in secretory IgA production and RegIII-*γ* antimicrobial peptide expression, thereby intensifying the ecological vacuum created by commensal depletion (Yuan et al., 2021). The recovery trajectory of CR after antibiotic discontinuation is often lengthy and extremely individualised, with some taxa unable to re-establish for months or even years after a single treatment course (Pang et al., 2019). A seminal longitudinal metagenomic study demonstrated that healthy adults receiving ciprofloxacin experienced rapid and severe reductions in microbial diversity within days of treatment initiation. Even after 1 year of follow-up, lasting disruptions in the microbial community were still observed, including a continued reduction in butyrate-producing bacteria (Dethlefsen et al., 2008; Dethlefsen and Relman, 2011). Patients with repeated histories of antibiotic courses tend to have more severe and prolonged dysbiosis with each subsequent treatment course. It is consistent with the ratchet -like progression loss in community diversity and CR capability imposed by serial antibiotic exposures (Dethlefsen and Relman, 2011). Because prolonged post-antibiotic periods of CR impairment create sustained ecological opportunities for resistant organisms to proliferate within the intestinal reservoir and disseminate locally or systemically, these disruptions have major implications for antimicrobial resistance development and transmission (de la Fuente-Nunez et al., 2023).

### 6.3 Pathogen expansion following CR loss

*Clostridium difficile* infection (CDI), which is the most common cause of healthcare-associated infections worldwide and is precipitated by prior exposure to antibiotics is the most clinically significant form of antibiotic-induced CR failure (Shivashankar et al., 2013). In animal models, prior exposure to antibiotics increases susceptibility to recurrent CDI, as it leads to a depletion of secondary bile acids and the anaerobic bacteria responsible for their production. Importantly, this susceptibility can be reversed through restoration of specific commensal organisms capable of regenerating these protective metabolites. The strongest clinical evidence supporting the concept that restoration of colonisation resistance, rather than direct antimicrobial activity alone, represents the critical therapeutic mechanism comes from faecal microbiota transplantation (FMT). By replenishing depleted microbial communities and restoring CR-associated metabolic functions, FMT achieves cure rates exceeding 85–90% in patients with recurrent CDI (Mamo et al., 2018). Under normal conditions, commensal microbiota typically suppresses multidrug-resistant organisms (MDROs) by competitive exclusion. However, antibiotic-induced loss of CR simultaneously permits intestinal domination by MDROs (Bharadwaj et al., 2022). In antibiotic-treated patients undergoing haematopoietic stem cell transplantation (HSCT), intestinal dominance defined as a single taxon making up more than 30% of the microbiota by vancomycin-resistant *Enterococcus* (VRE), *Klebsiella pneumoniae*, or *E. coli* strongly predicts subsequent MDRO bloodstream infection, indicating the gut as the main reservoir for systemic MDRO dissemination (Kim et al., 2019). The establishment of dense MDRO populations within the post-antibiotic gut environment also creates ideal conditions for horizontal gene transfer of mobile resistance elements between bacterial species, thereby amplifying the intestinal resistome and facilitating the spread of antimicrobial resistance (Lerner et al., 2017).

### 6.4 Antibiotic class-specific effects on CR

Different antibiotic classes produce qualitatively and quantitatively distinct patterns of CR disruption that reflect their antimicrobial spectra and modes of action (Fishbein et al., 2023). Fluoroquinolones and carbapenems, which achieve high luminal concentrations through intestinal and biliary secretion, have widespread disruptive effects on both Gram-positive and Gram-negative commensals, causing significant CR impairment and widespread ecological disruption (Rodrigues and Silva, 2025). The degree of CR disruption is correlated with anaerobic spectrum coverage, treatment duration, and mode of administration, according to a systematic review of 24 antibiotic classes. Oral and biliary-excreted parenteral agents cause significantly more collateral damage to the microbiome than systemic agents with minimal gut penetration. Clinical trials have demonstrated that the development of targeted “gut-sparing” antibiotic strategies, such as the use of oral activated charcoal adsorbents to capture residual luminal antibiotics, significantly reduces microbiome disruption without compromising systemic therapeutic efficacy, validating class-specific CR disruption as a modifiable exposure (Rai et al., 2025).

## 7 Host factors influencing microbiome-antibiotic-AMR outcomes

### 7.1 Age and developmental stage

The host age at the time of antibiotic exposure profoundly shapes the severity of microbiome disruption, the degree of resistome enrichment, and the capacity for community recovery reflecting fundamental differences in microbiome composition, immune maturation, and ecological stability across the human lifespan (Bradley and Haran, 2024). In infancy, the gut microbiome is characterised by low diversity, high temporal variability, and susceptibility to environmental perturbation, rendering it particularly vulnerable to antibiotic disruption during the critical development window in which commensal colonisation programs mucosal immunity and metabolic development (Bradley and Haran, 2024). A large randomised trial involving 147 infants demonstrated that antibiotic treatment not only acutely reduced microbiota diversity but also significantly enriched the resistome by increasing the abundance of mobile antibiotic resistance genes. Importantly, these resistome perturbations persisted for months after antibiotic cessation (Reyman et al., 2022). A complementary metagenomics study comparing 662 antibiotic-treated infants and 217 adults revealed that infants and adults exhibit fundamentally different resistome dynamics. Antibiotic treatment enriched mobile resistance genes more markedly in infants, whereas the duration of microbiome disruption was paradoxically longer in adults, indicating that the age-specific risks are bidirectional and clinically distinct (Li et al., 2023b). On the other end of the age spectrum, ageing is linked to a gradual decline in the diversity of gut microbes, a relative increase in Proteobacteria and pathobionts, and a decrease in butyrate-producing Firmicutes. These changes collectively constitute age-related baseline dysbiosis, which is independent of antibiotic exposure (Bradley and Haran, 2024). Elderly people living in long-term care facilities exhibit significant microbiome impoverishment correlated with dietary restriction, polypharmacy, and repeated antibiotic courses (Haran et al., 2021). Because of this age-related baseline dysbiosis, there is less ecological redundancy available to protect against antibiotic-mediated disruption. As a result, older patients experience more severe and prolonged impairment of CR with each subsequent antibiotic course compared to younger patients with similar pre-treatment diversity (Bradley and Haran, 2024). The elderly have a high-risk convergence for multidrug-resistant organism intestinal dominance and subsequent systemic dissemination after antibiotic treatment. This increased susceptibility is driven by impaired innate immune responses, increased mucosal permeability, and microbiome depletion, resulting in a substantially greater risk compared with younger adults receiving similar antibiotic regimens (Garcia et al., 2022).

### 7.2 Diet and nutritional status

Diet constitutes one of the most powerful modifiable host factors shaping the gut microbiome composition, metabolic function, and capacity for CR maintenance, with dietary fibre intake playing a particularly critical role in sustaining the anaerobic communities essential for CR (Makki et al., 2018). A seminal mechanistic study showed that the dietary environment plays a fundamental role in post-antibiotic microbiota recovery. Animals maintained on low-fibre diets showed persistent suppression of CR-mediating taxa for weeks after stopping antibiotics, while high-fibre supplementation sped up community restoration and CR recovery (Ng et al., 2019). The concept that dietary modulation of microbiome composition has population-level consequences for AMR dissemination is further supported by studies of ancestral gut microbiome, which are characterised by greater diversity and a higher Firmicutes abundance in populations maintaining traditional high-fibre diets. In comparison to westernised industrial populations, the ancestral gut microbiome exhibits significantly more robust CR and reduced resistome burden (Makki et al., 2018). Malnutrition reduces the quantity of fermentable substrates available for the recovery of commensal populations following the intake of CR-disrupting drugs, thereby further adversely affecting the resilience of the microbiome after exposure to antibiotics. Malnutrition is prevalent in low- and middle-income countries, as well as among hospitalised critically ill patients (Subramanian et al., 2014).

The interaction between polypharmacy, diet and the gut microbiome further modulates antibiotic outcomes in clinically underappreciated ways (Ticinesi et al., 2017). A landmark pharmacometagenomic study of 1,883 individuals demonstrated that numerous non-antibiotic medications, including proton pump inhibitors, metformin, and laxatives, independently alter gut microbiome composition in ways that may either potentiate or attenuate the dysbiotic effects of co-administered antibiotics. These findings emphasise that antibiotic-microbiome interactions cannot be evaluated in isolation from the broader medication context (Ticinesi et al., 2017). Proton pump inhibitors, for example, reduce gastric acidity and thereby facilitate the survival of acid-sensitive bacteria in the upper gastrointestinal tract, increasing the pool of potential colonisers capable of exploiting post-antibiotic ecological niches in the lower gut (Sawaid et al., 2025). These diet–drug–microbiome interactions underline the need for a systems-level approach to antibiotic stewardship that accounts not only for antibiotic choice but also for the nutritional and pharmacological context in which treatment occurs (Fishbein et al., 2023).

### 7.3 Host genetics

Host genetic diversity also modulates the effective luminal antibiotic concentrations that drive dysbiosis and select for resistant organisms by influencing antibiotic pharmacokinetics and pharmacodynamics (Fishbein et al., 2023). Polymorphisms in cytochrome P450 enzymes, intestinal transporters, and hepatic conjugation pathways can alter drug metabolism, absorption, distribution, and biliary excretion rates. Consequently, individuals who are poor metabolisers of specific antibiotics, or who exhibit elevated biliary drug concentrations due to transporter variants, may experience more severe and prolonged gut microbiome disruption following antibiotic treatment compared with individuals who are normal metabolisers (Tomaszewski et al., 2008). The complex interplay among host genotype, early-life microbial acquisition, dietary environment, and cumulative antibiotic exposure represents a multifactorial quantitative trait whose complete understanding will require the integration of metagenomic, genomic, metabolomic, and clinical datasets from large perspective cohort studies (Azad et al., 2017).

### 7.4 Immune status and comorbidities

The host immune status profoundly determines the interplay between antibiotic-induced microbiome disruption and AMR dissemination, with immunocompromised populations experiencing the most devastating convergence of these processes (Khazi and Ahmad, 2025). In immunosuppressed conditions, the gut microbiota often serves as the primary, and sometimes the only, protective barrier against multidrug-resistant organism (MDRO) colonisation and systemic dissemination. When antibiotics remove this barrier, no alternative immune mechanism remains to prevent translocation across the compromised mucosal surface (Takiishi et al., 2017). Patients with chronic kidney disease exhibit distinct gut microbiome alterations characterised by increased abundances of urease-positive and ureolytic bacteria, depletion of butyrate-producing commensals, and enrichment of mobile resistance gene pools. Following antibiotic treatment, this baseline resistance burden increases significantly and has been linked to elevated rates of clinical infection as well as post-treatment colonisation by multidrug-resistant organisms (MDROs). The antibiotic-associated accumulation of ESBL-producing *Enterobacteriaceae* within the intestinal reservoir correlates strongly with the cumulative antibiotic burden. Furthermore, it predicts systemic infections involving identical resistant clones during disease flares in patients with Inflammatory Bowel Disease (IBD)—a population that frequently receives antibiotic courses for infectious complications and whose microbiomes are inherently dysbiotic. The use of proton pump inhibitors, which are particularly common in hospitalised patients with the aforementioned comorbidities, independently decreases gut microbial diversity and encourages the overgrowth of *Enterobacteriaceae*, thereby amplifying the dysbiotic effects of co-administered antibiotics and compromising colonisation resistance against nosocomial pathogens (Sawaid et al., 2025).

## 8 Tools and approaches to study microbiome and AMR

### 8.1 Culture-based methods and antimicrobial susceptibility testing

The investigation of microbiome-AMR interactions has historically been anchored in culture-based methodologies, which remain indispensable for phenotypic characterisation of antimicrobial resistance. Culture-Based methods, as recommended by the European Committee on Antimicrobial Susceptibility Testing (EUCAST) and the Clinical Laboratory Standards Institute (CLSI), are considered the gold standard for identification of AMR in gut microbiota. These includes disc diffusion assays and MIC testing following the selective isolation of organisms (Deshpande et al., 2025). Despite their foundational role, these traditional methods are limited by significant delays in reporting. Traditional AST methods, such as agar disc diffusion and broth microdilution sometime take 18 to 24 h or even longer to give results, which is often too slow for timely clinical decision making, where rapid therapeutic intervention is critical. In addition, these methods are unable to detect non-cultivable bacteria that may harbor clinically significant AMR genes, thereby underestimating the overall resistome burden (Ahmad et al., 2024). To address these temporal limitations, rapid phenotypic platforms such as the VITEK REVEAL and Selux NGP assays have been developed, enabling real-time MIC monitoring directly from positive blood cultures within a few hours. With the continued rise in antimicrobial resistance, these platforms are expected to incorporate a broader range of AMR genes and drug–bug combinations, including WHO priority pathogens such as cephalosporin-resistant *Neisseria gonorrhoeae* and macrolide-resistant *Streptococcus* species (Hattab et al., 2024). Nevertheless, non-culture-based approaches offer potential in reducing the limitations, delays and inaccuracies associated with conventional microbiological techniques, paving the way for more accurate identification of causative pathogens with simultaneous AMR profiling (Hassall et al., 2024).

### 8.2 PCR-based and targeted molecular approaches

Polymerase chain reaction (PCR)-based methods have emerged as rapid, sensitive and cost-effective complements to culture-based diagnostics for the targeted detection and quantification of specific ARGs in microbiome samples. Quantitative PCR (qPCR) allows for the precise enumeration of clinically important resistance determinants, including *mecA*, *blaKPC, blaNDM,* and *vanA,* directly from complex sample matrices without prior bacterial cultivation, making it particularly well-suited for large-scale epidemiological surveillance studies. To evaluate gene expression variations, real-time PCR and reverse transcription-PCR are currently employed in combination. Studies conducted using RT-PCR have indicated that bacterial pathogens and their associated AMR genes can be identified from clinical respiratory samples in a rapid, straightforward, and highly specific manner (Elbehiry et al., 2025b). However, a fundamental limitation of PCR-based approaches is that they can only detect ARGs for which primers have been designed, meaning novel or uncharacterised resistance determinants may remain undetected. Furthermore, these methods are insufficient for independently predicting antimicrobial susceptibility because resistance phenotypes often arise from a complex interaction of resistance genes, regulatory pathways, efflux systems, and chromosomal mutations that cannot be inferred from a single gene target alone (Ahmad et al., 2024). Consequently, qPCR is most effective as a targeted surveillance and screening tool when integrated with broader sequencing-based approaches, rather than as a standalone strategy for comprehensive resistome characterisation.

### 8.3 Next-generation sequencing: 16S rRNA and shotgun metagenomics

Next-generation sequencing (NGS) technologies have revolutionised the study of microbial communities and AMR by enabling high-throughput characterisation of microbiome composition, functional potential, and resistome dynamics with unprecedented sensitivity and resolution. Among these approaches, 16S rRNA amplicon sequencing remains one of the most widely used methods for microbial taxonomic profiling. In microbial ecology studies, the highly variable V4 region of the 16S rRNA gene is frequently targeted because of its strong discriminatory capacity for identifying bacterial taxa across diverse biological and environmental samples (Caporaso et al., 2012). Illumina platforms are widely employed for this purpose due to their high read depth, scalability, and relatively low sequencing error rates.

However, despite its cost-effectiveness and utility in community profiling, 16S rRNA amplicon sequencing has several limitations, including restricted taxonomic resolution below the species level, primer-associated amplification bias, and limited functional characterisation. In addition, methodological variability during DNA extraction, amplification, sequencing, and taxonomic classification can substantially influence microbiome profiles. For example, different DNA extraction protocols may produce markedly different microbial compositions, while amplification-associated biases exceeding 85% have been reported in certain samples (Brooks et al., 2015).

To overcome the functional limitations of targeted amplicon sequencing, whole-genome shotgun metagenomics (WGS) has emerged as a more comprehensive approach that sequences all genetic material within a sample without prior amplification. This strategy enables simultaneous characterisation of microbial taxonomy, metabolic pathways, virulence determinants, and ARGs. Shotgun metagenomics has been extensively applied to human faecal, animal, avian, and environmental samples, revealing complex interactions among microbial communities, pathogenic bacteria, virulence factors, and resistome architecture across different ecological niches (Napit et al., 2025). Compared with 16S rRNA sequencing, WGS provides substantially greater insight into microbial functional potential and resistance mechanisms. However, conventional short-read metagenomic sequencing still has limitations in accurately identifying the genomic locations of ARGs, their associated mobile genetic elements, and their bacterial host species because short reads often fail to span complete resistance regions or repetitive elements (Quince et al., 2017).

To address these challenges, third-generation sequencing technologies have increasingly been incorporated into microbiome and resistome studies. Long-read sequencing platforms developed by Oxford Nanopore Technologies and Pacific Biosciences generate long and ultra-long reads capable of spanning full-length 16S rRNA genes and extended genomic regions. These technologies improve species-level taxonomic identification and facilitate accurate characterisation of structural variants, plasmids, transposons, integrons, and other mobile genetic elements associated with AMR (Che et al., 2019; Santos et al., 2020). Consequently, long-read metagenomic sequencing provides a more comprehensive understanding of ARG abundance, ARG-carrying species, and resistance-associated genetic contexts, making it particularly valuable for tracking the dissemination and evolution of resistance determinants within complex microbial communities (Dai et al., 2022). Technological advances have also substantially improved sequencing accuracy, with newer ONT chemistries achieving Q20 or higher read quality and PacBio circular consensus sequencing (CCS) generating highly accurate HiFi reads with average quality scores approaching Q27 (Wenger et al., 2019; Ferguson et al., 2022). Nevertheless, long-read platforms, particularly ONT, may still exhibit lower base-calling accuracy compared with short-read sequencing technologies. Therefore, hybrid sequencing strategies integrating short-read and long-read data are increasingly being adopted to combine the high accuracy of short reads with the superior genomic continuity of long reads, resulting in improved genome assembly, resistome profiling, and identification of ARG-host associations (Bertrand et al., 2019; Zhao et al., 2023b).

Collectively, these sequencing technologies are increasingly being integrated in complementary workflows to provide a systems-level understanding of microbial ecology and antimicrobial resistance.

### 8.4 Functional metagenomics and resistome databases

Beyond descriptive sequencing, functional metagenomics provides a culture-independent platform for identifying novel ARGs by cloning environmental DNA into expression vectors and screening for resistance phenotypes. Recent studies using functional metagenomics have highlighted the unappreciated diversity of AMR genes within the human microbiome and have identified genes that were not been described previously. In contrast, targeted PCR-based approaches remain promising for detecting and quantifying known ARGs within microbial populations (Lin et al., 2015). The annotation of ARGs from metagenomic datasets is critically dependent on reference databases like the Comprehensive Antibiotic Resistance Databases (CARD), ResFinder, MEGARes, and AMRFinderPlus. MEGARes and AMR++, v3.0 represent updated and comprehensive repositories of AMR determinants. These databases have better software pipeline for classification using high-throughput sequencing, which provide a bioinformatic framework for resistome research (Bonin et al., 2023). However, the common features of these databases are that they can identify only those resistance determinants that have already been catalogued within their sequence repositories. Thus, novel resistance genes, whether acquired through HGT or arising *de novo* may completely evade detection. Furthermore, the resistome and mobilome can be solved at the cellular level with single-cell genomics. This process facilitates the identification of mobile genetic elements that are often overlooked by metagenomics. This represents a next-generation frontier in the field of resistome characterisation (Elbehiry et al., 2025a).

### 8.5 Artificial intelligence and machine learning in AMR research

The development of genomic, metagenomic and clinical datasets has laid a favourable foundation for the application of artificial intelligence (AI) and machine learning (ML) in AMR research. This introduces new dimensions of analytical capability that extend beyond traditional bioinformatics pipelines. AI and ML models can utilise various data sources like clinical information, genomic sequences, microbiome insights and epidemiological data for predicting AMR outbreaks and provide insights into the discovery of novel antimicrobials, the repurposing of existing drugs and combination therapy through the analysis of molecular structures (Bilal et al., 2025). In the context of resistome characterisation, deep learning models such as DeepARG and HMD-ARG have demonstrated the capacity to classify ARGs from metagenomic sequences with high accuracy, even for genes with limited homology to known database entries (Li et al., 2021). AI-driven approaches have also been increasingly applied in bacterial infection diagnostics, AMR surveillance, antimicrobial peptide discovery, and antibiotic development. Recent reviews have summarised the application of contemporary AI models across these domains while also discussing critical considerations and limitations associated with implementing AI in microbiology and clinical medicine (Arnold et al., 2025). ML models play a pivotal role in monitoring AMR by analysing variety of data to identify novel resistance trends and potential zones, thereby enabling public health agencies to combat outbreaks of resistant infections. Deep learning, as a subfield of ML, can accelerate the identification of chemicals within large chemical libraries to facilitate the antibiotic drug discovery (Li et al., 2024). In spite of this promise model interpretability still faces tremendous challenges. The heterogeneity of training data, geographic biases in sequencing datasets, and the integration of AI outputs into clinical workflows highlight the need for prospective validation studies before widespread deployment.

## 9 How to overcome challenges

### 9.1 Methodological standardisation and bioinformatics challenges

One of the most persistent obstacles to advancing microbiome-AMR research is the absence of standardised protocols governing critical pre-analytical and analytical steps, creating profound barriers to cross-study comparability and meta-analysis. To enable the exploration, expansion and extraction of insights from existing data, it is essential to integrate data confidentiality, geopolitical and cultural variation, surveillance gaps and system thinking combined with modelling (Arnold et al., 2024). Variability in DNA extraction kits, sequencing platform choice, amplicon region selection, bioinformatic pipeline selection and ARG abundance normalisation strategies introduces systematic differences between datasets that impede direct comparison. WGS and metagenomics are highly valuable for outbreak tracing and resistance surveillance. However, they are limited by cost, turnaround time and the lack of standardised bioinformatics pipelines, as highlighted in recent reports of WHO GLASS and EFSA/ECDC (Elbehiry et al., 2025a). Moreover, a substantial translational gap still exists between research findings and routine clinical microbiological practice.

Although national surveillance systems can benefit significantly from genomic data, which offers profound information into pathogen epidemiology and the rise of AMR strains. AMR surveillance currently still primarily relies on phenotypic characterisation and genomic surveillance via WGS. While genomic sequencing is essential for integrating phenotypic and epidemiological data, it is not yet capable of completely replacing traditional antimicrobial susceptibility testing (Bianconi et al., 2023). To bridge this translational gap, it will be essential to invest in the capabilities of bioinformatics, international standards for data sharing, and the regulatory harmonisation of genomic diagnostic workflows.

### 9.2 Equity gaps and surveillance in low and middle-income countries

The worldwide burden of AMR is predominantly high in low and middle-income countries (LMICs), yet these settings remain critically underrepresented in microbiome-AMR surveillance infrastructure. In sub-Saharan Africa and parts of Asia, fewer than 30% of healthcare centres have regular access to cultural and antimicrobial susceptibility testing facilities, primarily due to a shortage of reagents, lack of staff, infrastructure constraints and weak quality systems (Elbehiry et al., 2025a). This display is not merely logistical; it creates critical blind spots in global resistome maps, as AMR hotspots in LMICs driven by high antibiotics consumption, poor sanitation and dense animal-human interfaces remain poorly characterised by sequence-based surveillance. The emergence of AMR poses a considerable risk and antimicrobial stewardship programs face context-specific implementation challenges that must account for disparities between high- and low-income settings, including governance, financial limitations, digital innovation gaps and sociocultural and behavioural contributors of antimicrobial use (James et al., 2026). Overcoming these equity gaps will require targeted investment in laboratory infrastructure, surveillance systems, workforce training, and capacity-building programs for resource-limited settings. By aligning political commitment, funding and scientific innovation, antimicrobial stewardship programs can be scaled effectively to preserve antimicrobial efficacy, mitigate AMR, improve health outcomes and promote global health security (James et al., 2026).

### 9.3 Horizontal gene transfer and the complexity of the gut resistome

A fundamental biological challenge in understanding and controlling AMR within the microbiome is the extraordinary complexity of HGT dynamics, through which resistance genes spread laterally across phylogenetically diverse bacteria via conjugation, transformation and transduction. Environmental factors such as availability of nutrient, pH, temperature, and exposure to drugs, influence the rate of conjugative plasmid transfer. Antibiotic-induced dysbiosis can also promote the transfer of MDR IncA/C plasmids into commensal *E. coli*, while also regulating the colonisation of *Salmonella* species in the gut (Deshpande et al., 2025). Resistance mechanisms operate across multiple biological levels, ranging from individual bacterial cells to entire microbial communities. Furthermore, during biofilm formation, the genomic potential for resistance can increase substantially through enhanced horizontal gene transfer, leading to the emergence of bacterial subpopulations capable of persisting under antibiotic exposure and other cellular stress conditions (Li et al., 2024).

The ecological complexity of the gut microbiome further complicates AMR tracking. The same ARG may be carried simultaneously by multiple taxa, transferred between plasmids and chromosomes, and maintained in both transcriptionally active and dormant states, all of which are difficult to capture using a single methodological approach. Therefore, understanding and disrupting HGT networks remains a critical research priority, requiring the development of mobile genetic element tracking tools and longitudinal resistome surveillance studies.

## 10 Microbiome-targeted therapeutic strategies

**Figure 3:**
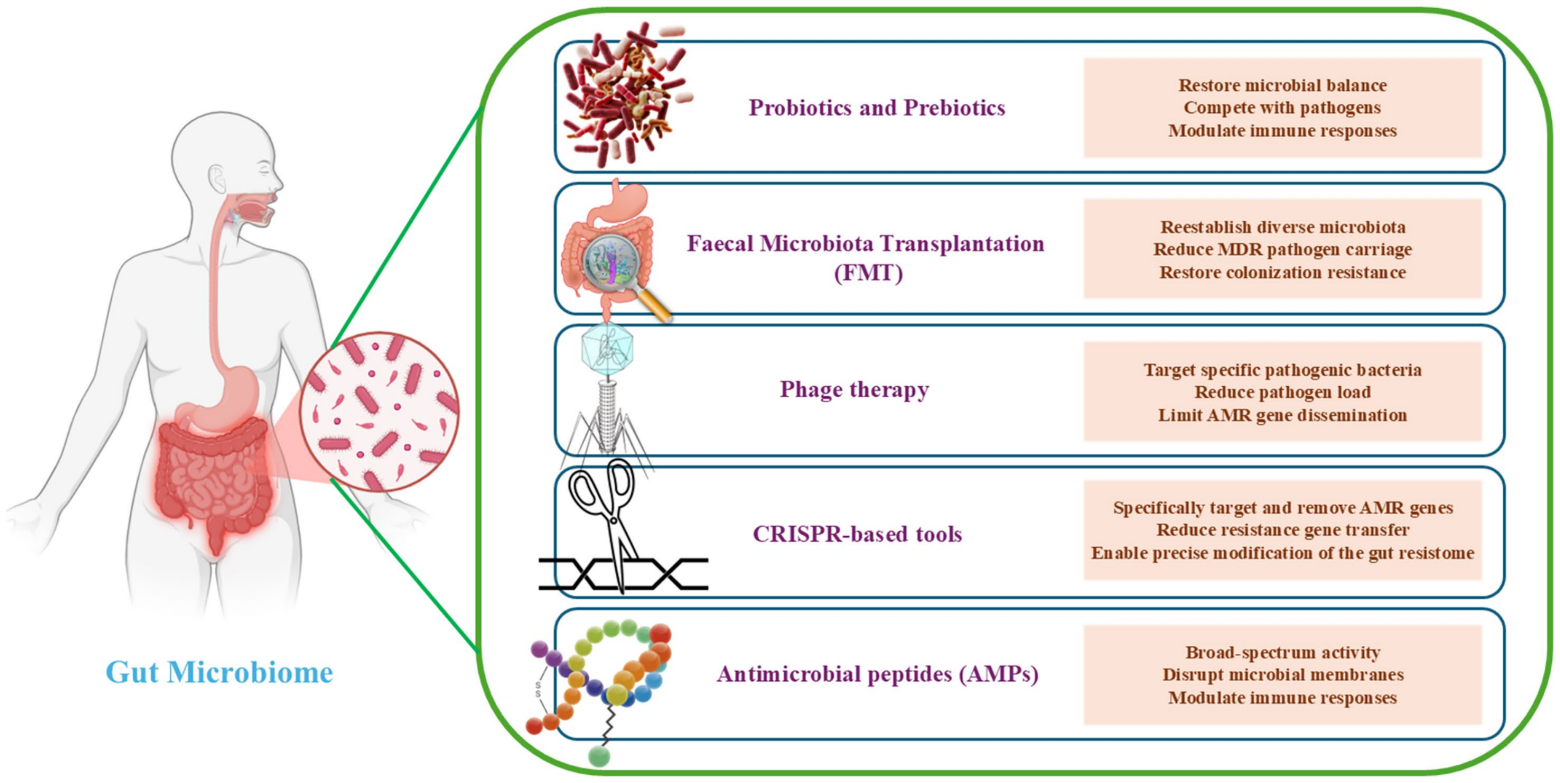
Microbiome-targeted therapeutic strategies to combat antimicrobial resistance. The figure illustrates emerging microbiome-based approaches for combating AMR and restoring gut microbial homeostasis.

Microbiome targeted therapeutic strategies are treatments designed to modify or restore gut microbiome so that we can improve health of individuals and treat disease (Figure 3).

### 10.1 Probiotics and prebiotics

Probiotics defined as live microorganisms that, when administered in adequate amounts, confer a health benefit on the host have been extensively investigated as adjunctive interventions to mitigate antibiotic-induced dysbiosis and curtail the expansion of ARGs within the gut resistome. Probiotic formulations of *Lactobacillus acidophilus*, *Bifidobacterium lactis*, and *Bifidobacterium longum* have been shown to maintain diversity of microbes during antibiotic therapy. It also reduces the elevated levels of ARGs following. This suggests that probiotics can act not only as symptom-management tools but also as ecological stabilisers that limit the selective advantage conferred on resistant organisms by antibiotic pressure (John et al., 2024).

Prebiotics are non-digestible dietary substrates such as inulin, fructooligosaccharides (FOS), and galactooligosaccharides (GOS) function as selective growth substrates for beneficial commensal bacteria and represent an emerging complementary strategy to combat AMR. Prebiotic fermentation by commensal *Bifidobacterium* and *Lactobacillus* species produces SCFAs, particularly acetate, propionate, and butyrate, which lower luminal pH and thereby suppress the proliferation of multidrug-resistant pathogens such as ESBL-producing *E. coli* (Thulasinathan et al., 2025). Notably, inulin combined with pantoprazole has been shown in preclinical studies to reduce intestinal carriage of multidrug-resistant *E. coli* through enhanced microbial propionate production, demonstrating the potential of prebiotic-based interventions for targeted MDRO decolonisation (Ishnaiwer et al., 2024).

### 10.2 Faecal microbiota transplantation (FMT)

Faecal microbiota transplantation (FMT) involves the transfer of a processed stool preparation from a healthy, screened donor into the gastrointestinal tract of a dysbiotic recipient, to restore a balanced microbial community and re-establish CR against pathogenic and resistant organisms. FMT has achieved its most robust clinical evidence base in the treatment of recurrent *Clostridioides difficile* infection (rCDI), where it demonstrates cure rates exceeding 85–90%, far surpassing those of repeated antibiotic courses, and led to the Food and Drug Administration’s (FDA) approval of the first live biotherapeutic products (Rebyota and Vowst) in 2022–2023 (Yadegar et al., 2024). Preclinical studies using animal models have provided important insights into the role of FMT in restoring CR against MDR organisms. In antibiotic treated mice colonised with vancomycin-resistant *Enterococcus* (VRE), donor microbiota enriched with Barnesiella spp. was shown to promote clearance of intestinal VRE colonisation (Ubeda et al., 2013; Caballero et al., 2017). Studies involving carbapenem-resistant *Klebsiella pneumoniae* (CRKP) demonstrated that restoration of a healthy gut microbiota through FMT significantly reduced the intestinal burden of the pathogen (Osbelt et al., 2021). Although serious adverse effects may be underreported, FMT is generally considered safe in immunocompetent individuals, with only infrequent reports of complications such as hospitalisation, pathogen transmission, or death.

Despite its potential therapeutic benefits, FMT remains a relatively non-standardised and partially regulated strategy, with persistent concerns regarding safety and long-term outcomes (Nicholson et al., 2020) reported that the most common adverse events observed in children undergoing FMT included diarrhea, abdominal pain, and bloating. Furthermore, concerns have been raised regarding the potential transmission of MDR pathogens through donor stool. The U. S. FDA issued safety alerts following reports of infections caused by extended-spectrum *β*-lactamase (ESBL)-producing *E. coli* transmitted via FMT, including one fatal case in immunocompromised patients [U.S. Food and Drug Administration (FDA), 2020]. These incidents underscored the importance of rigorous donor selection, comprehensive microbiological screening, and standardised safety protocols prior to administering FMT (DeFilipp et al., 2019). Consequently, current guidelines recommend enhanced screening for MDR organisms and other transmissible pathogens to mitigate the risk of adverse events associated with FMT therapy (Cammarota et al., 2019).

### 10.3 Phage therapy

Bacteriophage (phage) therapy involves the use of naturally occurring or engineered viruses that selectively infect bacterial hosts. It is highly targeted approach for the eradication of antibiotic-resistant bacteria and avoid the collateral damage to the commensal microbiome that is characteristic of traditional antibiotic therapy. Unlike antibiotics, phages display a limited host-range, typically infecting only a single bacterial species or specific subset of strains within a species. Thus, they selectively destroy the pathogenic target while preserving the ecological integrity of the surrounding microbial community (Mmbando et al., 2025). Recent clinical and preclinical developments have suggested the feasibility of phage-based approaches. Engineered phage cocktails targeting multidrug-resistant *E. coli*, *K. pneumoniae*, and *P. aeruginosa* have proven effective in compassionate-use case series and early-phase clinical trials. In these trials, the administration of phages resulted in a significant reduction in the burden of the targeted pathogens, accompanied by minimal side effects (Chigozie et al., 2025).

### 10.4 CRISPR-based tools

The Clustered Regularly Interspaced Short Palindromic Repeats (CRISPR)-Cas systems are the most revolutionary platform for precision microbiome engineering. It is capable of targeting and killing particular antibiotic resistance genes or bacterial strains present within complex microbial communities, without disrupting the broader commensal ecosystem. CRISPR-based antimicrobials function by programming guide RNAs to direct the Cas nuclease to specific DNA sequences such as ARGs on plasmids or essential chromosomal loci in target pathogens resulting in sequence-specific DNA cleavage that either kills the target cell or cures it of its resistance-conferring genetic elements (Brödel et al., 2024). The CoCas9 variant, identified from the human microbiome, has emerged as a promising tool for microbial genome editing. Compared with conventional Cas9 proteins, CoCas9 is relatively compact, consisting of 1,004 amino acids, which may facilitate its delivery and application in diverse system (Pedrazzoli et al., 2024).

### 10.5 Antimicrobial peptides

Antimicrobial peptides (AMPs) are emerging as promising microbiome-targeted therapeutics with potential applications in gut-brain axis disorders (Sharma et al., 2025). AMPs are small cationic peptides naturally produced by animals, plants, and microorganisms as part of the innate immune defence system. Owing to their broad-spectrum antimicrobial activity, immunomodulatory properties, and lower propensity for resistance development, they are increasingly being explored as alternatives to conventional antibiotics (Mba and Nweze, 2022). Several AMPs are currently under clinical evaluation for infectious and inflammatory diseases (Dijksteel et al., 2021). Furthermore, the use of computational methods such as molecular docking, molecular dynamics simulations, and ML assisted screening to identify and optimise AMPs against MDR and XDR pathogens is rapidly increasing (Naha and Ramaiah, 2024; George et al., 2025).

Host-derived peptides such as LL-37 and defensins, as well as microbial-derived bacteriocins including nisin, have demonstrated the ability to inhibit multidrug-resistant pathogens while preserving beneficial gut microbiota. In addition to direct antimicrobial activity, these peptides contribute to maintenance of intestinal barrier integrity, modulation of inflammatory responses, and regulation of host immune signaling. Recent studies suggest that AMPs may help reduce intestinal reservoirs of MDROs with comparatively limited disruption of commensal microbial communities (Gong et al., 2021).

AMPs are also being investigated as potential agents for gut decolonisation of resistant bacteria. Although clinical evidence remains limited, several preclinical studies have reported encouraging findings. Bacteriocins, a group of ribosomally synthesised antimicrobial peptides produced by bacteria, have attracted considerable attention as potential substitutes for traditional antibiotics. In a mouse model, colonisation with bacteria producing bacteriocin-21 successfully eliminated intestinal vancomycin-resistant *Enterococcus* (VRE) colonisation without significantly altering the native gut microbiota (Kommineni et al., 2015). These findings highlight the therapeutic potential of bacteriocins and other AMPs in selectively targeting resistant pathogens within the gut environment.

Recent advances have focused on engineered AMPs, synthetic peptide optimisation, and probiotic-based delivery systems designed to improve peptide stability and targeted activity within the gastrointestinal tract. Emerging approaches such as engineered probiotic “peptide factories” and phage-AMP conjugates are being developed to achieve precise eradication of MDROs while minimising collateral microbiome disruption (Zhao et al., 2023a; Sharma et al., 2025). Collectively, these advances position AMPs as promising next-generation therapeutics for managing antimicrobial resistance and microbiome-associated gut–brain axis disorders.

## 11 One health approaches and global policy framework

Sustainably addressing the intersection of microbiome disruption and AMR requires moving beyond an individual-patient or single-institution paradigm toward integrated One Health frameworks that connect human, animal, agricultural and environmental surveillance systems. AMR is a multifaceted One Health challenge that affects humans, animals, plants and the environment, with more impacts on population health, food security and global economies.

A major driver of this crisis is the extensive use of antimicrobials in intensive livestock production systems (Matheou et al., 2025). It has been estimated that the majority of medically important antibiotics produced globally are administered to livestock, often for non-therapeutic purposes such as disease prevention and growth promotion. Globally, antimicrobial consumption in livestock is predicted to increase by more than 67% by 2030, rising from approximately 63,000 tons in 2010 to over 105,000 tons, primarily due to the growing demand for animal-derived food products and intensification of farming practices (Mehdi et al., 2018).

The widespread and prolonged use of antibiotics in food animals creates strong selective pressure for the emergence and dissemination of antibiotic-resistant bacteria (ARB) and ARGs within agricultural ecosystems. Importantly, a substantial proportion of administered antibiotics are excreted unmetabolised, leading to the accumulation of antibiotic residues, resistant bacteria, and ARGs in manure, agricultural soils, wastewater, and aquatic environments. The continuous release and persistence of these compounds contribute to ecological disturbances and promote the expansion of agricultural and environmental resistomes (Marshall and Levy, 2011; Hernando-Amado et al., 2019). Livestock-associated resistant bacteria and ARGs can subsequently spread to humans through contaminated food products, agricultural runoff, irrigation systems, direct animal contact, water sources, and the use of manure as fertiliser. Indeed, resistant bacteria have been detected throughout multiple stages of food production and processing, highlighting the important role of animal-associated reservoirs in the transmission and global dissemination of AMR (Witte, 2000; Chang et al., 2015).

Addressing this growing threat therefore requires coordinated stewardship at multiple levels. These efforts should encompass all stages of the antimicrobial lifecycle, including research and development, regulatory approval, production, selection, appropriate use, and safe disposal of antimicrobials (Altevogt et al., 2025). ARGs circulate dynamically across human, livestock and environmental compartments, particularly through agricultural runoff, wastewater discharge, contaminated soil and water systems and international trade in food animals. Consequently, resistance emerging in veterinary or environmental settings can re-enter and persist within human-associated microbiomes, thereby contributing to the continued spread of AMR. In this context, Innovative computational methods integrating big data streams from clinical, agricultural and environmental monitoring will accelerate understanding of AMR, supporting decision making. However, major challenges remain in the collection, harmonisation, integration, interpretation, and standardisation of these highly complex and multidimensional datasets (Arnold et al., 2024).

The long-term success of antimicrobial stewardship programs will also depend on strengthening governance, sustainable financing, digital innovation, surveillance infrastructure, and behavioural science–driven interventions. Importantly, stewardship policies must move beyond healthcare settings to incorporate stronger participation from agricultural, veterinary, food production, and environmental sectors, thereby enabling a more comprehensive and sustainable response to AMR at the global level (James et al., 2026).

## 12 Future directions

Future research on microbiome driven antibiotic resistance requires high resolution, longitudinal analysis of the gut resistome across diverse developmental stages and clinical settings, for better understand ARG behavior, long term studies integrating metagenomics, mobilome profiling and clinical data across infants, adults and high risk patients conditions are required to map ARG dynamics and relate them to antibiotic exposure and patient outcomes (Willmann et al., 2019). Experimental systems that integrate humanised microbiota with ARG tracking approaches like fluorescent tagging and Hi-C and experimental systems can help map conjugation networks, detect key microbial players and determine conditions that regulate horizontal gene transfer and integron-associated ARG acquisition (Kent et al., 2020).

Further work should also focus on identifying host, environmental and therapeutic factors that influence antibiotic-driven AMR, as recent datasets emphasising the importance of age, microbiome development, diet and ecological interactions in shaping resistome dynamics (Xu et al., 2024). Targeted microbiome-based interventions must be systematically evaluated for their ability to reduce ARG burden without unintentionally promoting resistant strains, plasmid dissemination, or ecological instability within the gut microbiota (Casaburi et al., 2019).

The shift toward precision stewardship will rely on models that use baseline microbiome and host factors to forecast resistome changes under different antibiotic treatments, supporting individualised and resistance-aware antibiotic use (Li et al., 2019). Incorporating metagenomic resistome monitoring into clinical trials and high-risk patient populations may enable evidence-based policies to control ARG amplification and limit downstream spread (Kiu et al., 2025).

A major future direction involves translating microbiome-targeted therapeutics into clinical applications. Engineered bacteriophages and phage cocktails are increasingly being explored as precision therapies capable of selectively eliminating MDR pathogens from the gut microbiome. Recent studies have demonstrated that CRISPR-Cas equipped bacteriophages can specifically target and cleave resistance determinants, thereby restoring bacterial susceptibility to conventional antibiotics. However, successful clinical translation will require robust trials that evaluate efficacy, safety, pharmacokinetics, improved delivery systems, and rapid companion diagnostics for pathogen identification (Wang et al., 2025; Piracha and Saeed, 2026).

CRISPR-based antimicrobial technologies also represent a critical area for future research. Sequence-specific CRISPR-Cas systems delivered via bacteriophages, conjugative plasmids, or engineered probiotics can enable the selective removal of ARGs within the gut or the targeted elimination of resistant bacterial populations. Emerging “living therapeutics,” including engineered commensal bacteria designed to secrete AMPs, quorum-sensing inhibitors, or CRISPR payloads, offer promising next-generation strategies for microbiome modulation. Nevertheless, additional *in vivo* studies are required to evaluate their colonisation efficiency, stability, long-term ecological impact, and host safety (Amen et al., 2025).

Advancements in synthetic biology could further enable rational microbiome engineering approaches aimed at combating AMR. These include the construction of beneficial bacteria capable of degrading residual antibiotics, disrupting biofilms, modulating inflammatory processes, or generating narrow-spectrum AMPs that selectively target MDR organisms while preserving beneficial commensals. Combination approaches such as phage-AMP therapy, synbiotics, and microbiome restoration strategies may offer synergistic effects for the eradication of resistant pathogens and the restoration of gut microbial homeostasis (Sharma et al., 2025).

Future research should focus on translational approaches that integrate microbiology, systems biology, synthetic biology, AI, and clinical medicine to accelerate the development of personalised microbiome therapies and improve understanding of host–microbe interaction networks associated with antimicrobial resistance and microbiome-associated diseases (Miryala and Ramaiah, 2019; Miryala et al., 2021). For successful clinical translation against antimicrobial resistance, establishing regulatory and safety frameworks for phage therapy, CRISPR-based therapies, and microbiome engineering will be imperative.

## 13 Conclusion

In this review, we highlighted the role of the gut microbiome in the development and dissemination of AMR. Although antibiotics remain indispensable in clinical practice, their use can also induce dysbiosis, reduce microbial diversity, and impair colonisation resistance, thereby promoting expansion of the intestinal resistome. The interconnected mechanisms driving the evolution of AMR within the gut include horizontal gene transfer, mobilisation of genetic elements, and bacterial adaptation. Consequently, the gut microbiome serves as a major reservoir of resistance genes, impacting not only individual health but also the global burden of infectious diseases. These interactions are further modulated by host-associated factors such as age, diet, immune status, genetics, and environmental influences, highlighting the need for both systems-level and personalised approaches to AMR management. Importantly, microbiome preservation remains an underrecognised aspect of current antimicrobial stewardship initiatives. Emerging microbiome-targeted interventions, including probiotics, prebiotics, faecal microbiota transplantation, bacteriophage therapy, antimicrobial peptides and CRISPR-based interventions hold promise as strategies for mitigating AMR. Nevertheless, challenges persist, such as the inherent complexity of the ecosystem, inadequate surveillance, and a lack of standardisation. Future efforts should therefore focus on integrating multi-omics, artificial intelligence, and the ‘One Health’ framework to improve the surveillance and management of AMR. Ultimately, transitioning toward microbiome-aware antimicrobial stewardship will be essential for preserving antibiotic efficacy, limiting the spread of resistance, and safeguarding global public health.
